# A report of a South African university’s management of undergraduate nursing students’ teaching and learning following the COVID-19 interruptions

**DOI:** 10.4102/hsag.v27i0.1816

**Published:** 2022-03-31

**Authors:** Olivia B. Baloyi, Mary Ann Jarvis, Ntombifikile G. Mtshali

**Affiliations:** 1 Discipline of Nursing, College of Health Sciences, University of KwaZulu-Natal, Durban, South Africa

**Keywords:** COVID-19, Donabedian, online teaching and learning, transition, undergraduate nursing students

## Abstract

The coronavirus disease 2019 (COVID-19) pandemic exposed an absence of blueprints to avert an education disaster. In South Africa, in line with Alert Level-5, adhering to lockdown restrictions, higher education institutions (HEIs) closed, necessitating the transition to online teaching and learning. The HEIs, inclusive of the nursing discipline, needed to develop comprehensive plans and a rigorous follow-up scheme in order to ensure that faculty and students made proper use of virtual platforms and simultaneously met regulatory body requirements, thus ensuring that ‘no student and faculty were left behind’. The responses varied from one HEI to another. The objective of this study was to present how a South African nursing education faculty managed teaching and learning following COVID-19-related interruptions. This included an HEI in KwaZulu-Natal, South Africa. Donabedian’s tripartite model, comprising structure, process and outcome, provides the organising structure to present the faculty and university’s approach to meet the desired outcome of saving the 2020 academic year. The Structures’ and Processes’ components of Donabedian’s tripartite model influenced both intended and unintended outcomes. In 3 months, what might have been argued as impossible, a 4-year undergraduate nursing programme was transitioned from a traditional approach to fully virtual remote teaching and learning. Thus, the 2020 academic year was saved.

**Contribution:**

This article offers guidance to HEIs on how to continue teaching and learning in contexts where education is interrupted.

## Introduction and background

As early as 1948, through the establishment of the World Health Organisation (WHO), measures were in place to further international cooperation for improved public health conditions (WHO n.d.), facilitating the declaration on 11 March 2020, of the coronavirus disease as a pandemic (WHO 2020). However, no similar global body is directed towards education or, more specifically, higher education, leaving each country without a blueprint to avert an education disaster. In South Africa, soon after the announcement of the pandemic (WHO 2020), on 15 March, 2020, the president declared a state of disaster, placing the country in a national shutdown from 26 March, 2020 (SA Government 2020a). Following the President’s announcement on the ban of large gatherings, in line with Alert Level-5 (SA Government 2020a), the Minister of Higher Education and Training declared on 17 March, 2020, that higher education institutions (HEIs) were to close for an early recess (Motala & Menon 2020). The early closure allowed preparation time as no disaster plans were in place to mitigate the impact of a pandemic on higher education.

Face-to-face contact classes with the students were suspended and left education institutions unprepared for the abrupt interruption in teaching and learning to weigh the value of education against the risk of exposing the students to the virus (Jackson et al. 2020). Fears of losing part of the academic year, with professional programmes failing to meet the regulatory bodies’ clinical requirements, raised concerns (Dewart et al. 2020). The pandemic had a strong and rapid impact on educational institutions, which led to a drastic change in the delivery approach of education (Johnson, Veletsianos & Seaman 2020).

Most institutions of higher learning converted to virtual remote learning through e-learning platforms, combining asynchronous and synchronous teaching (Knie et al. 2020) in order to ensure that teaching and learning continued while adhering to lockdown restrictions (Agu, Mcfarlane-Stewart & Rae 2021). The abrupt migration ensured that students did not lose more academic time, and thus, final-year students had an opportunity to complete their studies (Agu et al. 2021). However, HEIs needed to develop comprehensive plans and a rigorous follow-up scheme in order to ensure that faculty and students properly use e-learning platforms (Monareng, Ramraj & Mashau 2020; Tamrat 2020; Tamrat & Teferra 2020). Institutions managed to speed up the adoption and use of innovative online teaching and learning strategies, building the capacity of educators and students in this skill area and mobilising relevant resources for support (Agu et al. 2021; Jackson et al. 2020).

Despite the successful experiences of many universities in higher income countries, migrating from traditional teaching and learning to entirely online platforms imposed challenges to nursing students, particularly those in lower-middle-income countries, such as South Africa (Jamshidi et al. 2016). In nursing education, not only did the theoretical component pose delivery challenges but also exposed a gap for clinical teaching, as both components are required to complete the qualification (Aslan & Pekince 2021; Car et al. 2019). Nursing education can be understood as competency-based learning. Clinical exposure allows the student to exercise higher order thinking skills as theory is applied to the authentic clinical setting (Baloyi & Mtshali, 2018). Applying higher order thinking skills equips nurses and midwives with knowledge, skills and attitudes to execute the duties required in holistic nursing and midwifery care. Most of the teaching and learning provided to nursing and midwifery students occurs in a clinical environment (Jamshidi et al. 2016), guided by regulatory bodies. The South African Nursing Council (SANC), as the regulatory body, provided guidance, as did HEIs on how best to ensure relevant and responsive nursing graduates.

## Methodology

### Aim

The responses and challenges varied between HEIs, driving the aim of this study. The aim was to present a retrospective report on how the nursing education faculty within a HEI in Kwazulu-Natal, South Africa managed teaching and learning following COVID-19-related interruptions and offer insight for establishing guidelines directed to the process.

### Guiding framework

Donabedian’s tripartite model, comprising Structure, Process and Outcome, provides the organising structure to present the faculty and university’s approach to meet the desired outcome (Ameh et al. 2017). This article assumes that an established structure is a prerequisite to an effective process, and effective processes increase the likelihood of desired quality outcomes (Ameh et al. 2017).

Avedis Donabedian originally designed his tripartite model to provide structure to evaluate the quality of delivery of healthcare services, which is drawn from three components of structure, process and outcome (Donabedian 2005). This article translates the original use of the model into the context of a HEI. Donabedian’s model has been applied in a South African HEI study when developing a theory-informed interprofessional programme in the health sciences (Botma & Labuschagne 2019). Although the researchers can draw parallels between their use of Donabedian’s model and that by Botma and Labuschagne (2019), the context and outcome make for differences.

### Data sources

Documents, specifically aimed at saving the 2020 academic year, served as data sources. Documents included inter alia guiding South African Government legislation and COVID-19 regulations, Department of Higher Education policies, SANC circulars, university-specific strategies, teaching and learning policies and discipline-specific records.

### Ethical considerations

The process of providing a document report linked to document did not require ethical approval; consequently, no faculty or students’ voices are presented in this report. This study followed all ethical standards for research without direct contact with human or animal subjects.

## Results

### Presenting institution’s response to COVID-19 interruptions

#### Institutional context

The nursing education institution under discussion is part of an HEI, offering undergraduate and postgraduate programmes. The four-year undergraduate nursing degree follows a problem-based, competency-orientated, student-centred curriculum (Mtshali & Gwele 2015). Pre-COVID-19, teaching and learning involved face-to-face contact and access to an e-learning platform, with sit-in examinations to assess the learning outcomes. The Discipline of Nursing draws most of its student population from under-resourced settings, predominantly rural areas in and out of the province (Mudaly & Mtshali 2018). Table 1 depicts the core information about the undergraduate nursing students (Year 1–4; *N* = 307) at the start of the COVID-19 pandemic, as the university transitioned to online teaching and learning, highlighting that nearly two-thirds of the students were from rural areas.

**TABLE 1:** Core information about undergraduate nursing students at the time of transition.

Year	Urban (U) or rural (R) base residence		No data required		Reliable internet connection		Available device	
	*n*	%	*n*	%	*n*	%	*n*	%
Year 1 (*n* = 80)	30 U	38	0	0	52	65	16	20
	50 R	62	-	-	-	-	-	-
Year 2 (*n* = 76)	32 U	42	5	8	36	47	53	70
	44 R	58	-	-	-	-	-	-
Year 3 (*n* = 76)	28 U	37	2	3	55	72	56	74
	48 R	63	-	-	-	-	-	-
Year 4 (*n* = 75)	25 U	33	0	0	34	45	41	55
	50 R	66	-	-	-	-	-	-

**Total (*N* = 307)**	**115 U**	**37**	**7**	**2**	**177**	**58**	**166**	**54**
	**192 R**	**63**	**-**	**-**	**-**	**-**	**-**	**-**

**FIGURE 1:**
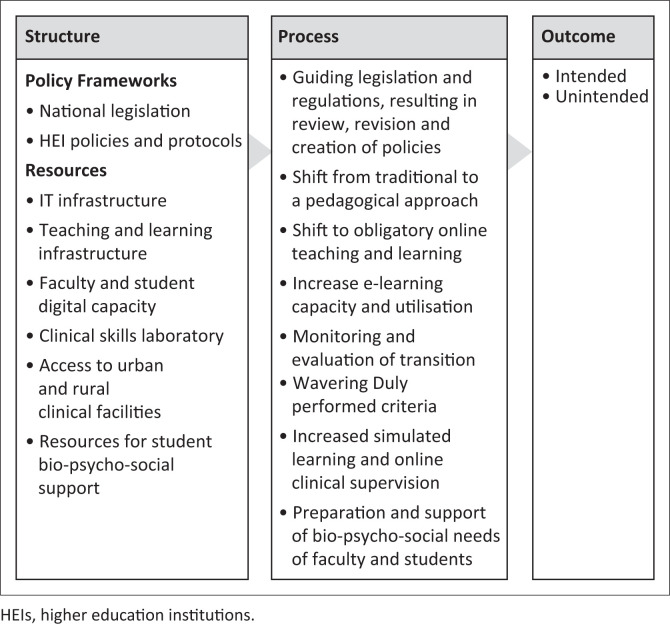
Higher education institutions’ response to interruptions to teaching and learning caused by COVID-19, framed within Donabedian’s tripartite model.

The desired outcome of Universities South Africa (USAf 2020) underpinned the institution’s processes, to save the 2020 academic year, and that no student or faculty should be left behind. Furthermore, the desired outcome focused on processes inherent in the faculty’s provision for and utilisation of structures to facilitate and support teaching, learning and assessment in response to the COVID-19-related intrusion and interruptions in education. The desired outcomes were achieved through structures and processes, consequently adopting Donabedian’s tripartite (2005) model as the article’s framework for the report on the transitioning to online teaching, learning and assessment in the select HEI (Figure 1).

### Structure component of Donabedian’s model applicable to the context

The structure component of Donabedian’s model in the context of this article focuses on the availability of legislation, policies or policy guidelines and resources (human, information and technology) *in situ* pre-COVID-19 and which are available to support the changes in response to the interruptions caused by the pandemic (Figure 1).

#### Policy frameworks

The Department of Higher Education and Training (DHET), the nursing regulatory body (SANC) and the university did not have a disaster management plan targeting an education crisis. The overarching *Disaster Management Act*, 2002 (Act No. 57 of 2002), with its subsequent regulations relating to COVID-19, provided the legislative framework for all decisions (RSA 2002). In the absence of existing institutional and regulatory body policy guidelines, the Teaching and Learning Committees of the HEI under discussion needed to draw their guidance from the *Disaster Management Act*, 2002 (RSA 2002) while maintaining their respective professional and educational standards.

#### IT infrastructure

For over a decade, the university had promoted e-learning initiatives, reflected through Goal-4 (Excellence in Teaching and Learning) of its earlier strategic plans (2007–2016) (UKZN 2017). Goal-4 required integrating IT networks and communication protocols into teaching and learning settings (UKZN 2017). The heightened IT focus ensured inter-alia efficient IT networks across the campuses and Virtual Private Network (VPN) connectivity for off-campus access of such resources as the library (UKZN ICS 2020).

#### Teaching and learning infrastructure

In addition, there existed a dedicated e-learning or online website, which allowed for multiple bi-directional teaching and learning interactions between the faculty and the learner. The university’s strategic plans (2017–2021) were translated into the policy on teaching and learning of quality online learning environments and resources in line with best practices in technology (UKZN 2017). However, pre-COVID-19, individuals or departments exercised the option to use the website (UKZN 2017) instead of the non-optional requirement of all faculties imposed by the current pandemic. Teaching and learning infrastructures also included well-equipped functional clinical skills laboratories with relevant technology, inclusive of high-fidelity manikins to simulate real-life situations, to meet the clinical component of the nursing programme. Further complimenting the simulated clinical resources, there was an established access to real-life authentic clinical learning in rural and urban settings.

#### Faculty and student digital capacity

There was a wide variance in digital literacy and access to the e-learning website among the students and faculty. The variance between the digital literacy of the first year and final-year undergraduate students (mean age 20.5 years) can be attributed in part to the shift in student enrolments from lower quintile schools (UKZN 2019), accommodating pupils from lower resourced settings who required upskilling. In particular, first-year students received computer literacy training. The students, except the first year students, had acquired the necessary hardware, laptops and related software through the years. Similarly, the faculties showed differences in their digital ability; hence, they were expected to attend the university technology enhanced learning (UTEL) programme designed for the attendee to embrace technology (UKZN 2019). Despite the opportunities, not all faculties attended the UTEL programme training, leaving a variance in digital capacity (UKZN 2019).

#### Resources for student bio-psycho-social needs

Not only was digital upskilling of importance but it gave recognition to the students’ bio-psycho-social needs. Student support services were available for psycho-social needs, while a campus clinic ensured the meeting of students’ physical health needs.

### Process component of Donabedian’s model applied to the transition

Process in Donabedian’s tripartite model focuses on procedures and logistics to support change (Ameh et al. 2017) and innovation guided by legislation, regulatory and institutional bodies. The process component of the model did not unfold in a stepwise manner but rather unfold in an iterative-staged approach (Figure 1).

#### Guiding legislation and regulations resulting in revision and creation of policies

The process was guided by the National Command Council and legislation (*Disaster Management Act* No. 57 of 2002 and regulations) (RSA 2002) and driven by the notion that teaching, learning and assessment should continue despite the pandemic (PMG 2020). In keeping with the goal of saving the academic year, The Department of Higher Education and Training Ministry charged HEIs with owning the process for a staggered return of students (PMG 2020). In particular, the legislated concession (Gazette No. 43 486) was given meaning through the DHET Risk Adjusted Strategy for the post-school education and training sector (PSET), which allowed for final-year health science students to return to campus, enabling them to meet their clinical requirements (SA Government 2020b). The regulatory body (SANC) accommodated for its insistence in the completion of the stipulated clinical hours (Circular 5/2020) (SANC 2020a) through a later circular by extending the training period and increasing simulation learning to 20% of the clinical hours (Circular 11/2020) (SANC 2020b). The process in the university aligned with the national desired outcome and the requirement of leaving no faculty or student behind, and accordingly developed a teaching and learning framework from the university disaster management plan, goaled at the recovery of the academic programme of 2020 (Songca 2020a). The Project Plan of Action for Remote Multi-Modal Teaching and Learning (2020) guided teaching, learning and assessment (Songca 2020b).

#### Shift from traditional teaching and learning to a pedagogical approach

The university embarked on alternative means of teaching and learning. Predominant use was made of the flipped classroom, a form of blended learning with content made available prior and after the online classes through various means, for example, triggers material involving case studies (Baloyi & Mtshali 2018). In addition, off-line material (preloaded flash drives, paper-based interactive learning content) was made available on request.

During the pandemic, *a shift to obligatory online teaching and learning* accompanied the move to a pedagogical approach. Initially, the interaction between the faculty and students employed various virtual platforms, for example, social media and dedicated e-learning websites. In most circumstances, limited student financial resources, unreliable network access (*n* = 130, 42%), or no device (*n* = 141, 46%) (Table 1) drove the realistic and relevant decisions on which platform to use. The interim choices bridged the gap until DHET had completed negotiations with mobile companies for reasonable data rates and network providers for zero-rating university-linked websites, thus enabling access to digital resources such as the online library (RSA 2002). The university mobilised the appropriate mobile resources and, every month, provided mobile data to each student (10GB per day, 30GB per night) and faculty (30GB per day, 80GB per night). Only 2% (*n* = 7) of the students did not require data (Table 1). Video-communication licenses were increased, faculties were provided with modems and funded students received laptops, while privately funded students received loans from the university to purchase laptops, in particular, to meet the deficit in first-year students (20% available device) (Table 1).

The move to a cyclical online learning model involved revising and approving faculty-specific and university teaching and learning structures, module guides, templates and clarifying learning outcomes to align with online learning standards and teaching strategies (Songca 2020b). Individual and virtual group engagement with the available learning resources and the practice of self-assessment ensured that learning was taking place while maintaining the quality (Songca 2020b). Continuous assessments replaced formative and summative assessments, retaining the clinical assessment at the insistence of the regulatory body (Circular 11/2020) (SANCb).

#### Increasing e-learning capacity and utilisation of faculty and students

The faculty and students were re-oriented through multiple workshops, by specialist divisions in the university, to the changed pedagogical approach. The pedagogy was not limited to but included faculty and learners’ upskilling on remote teaching and learning involving the flipped classroom and assessment strategies. The remote teaching tools used were online forum discussions, PowerPoint presentations with audio, recorded online lectures, WhatsApp audio messages and chats, distributed through inter-alia e-mails, e-learning platform and online video-conferencing service. Information and Communication Services managed a dedicated online expert technical support team for both software and hardware (Songca 2020b). The capacity building of faculty was not limited to within the university but through inter-institutional collaboration. Other HEIs developed repositories with ready-to-use content for faculty and students and, through collaboration, shared their resources. In addition, publishing houses shared access to their nursing education repository, inclusive of online courses for student learning skills and clinical competencies. Students developed competencies inter-alia in the skills needed for nursing during a pandemic, such as those linked to the efficient use of personal protective equipment (PPE).

As a platform to survey the teaching and learning issues, including *monitoring and evaluation*, the university timetabled a week for pilot testing the transitioning to online classes (dry-run), followed by a week of mock online assessments. These ring-fenced weeks served as a trial and provided information for relevant adjustments, ensuring no student was left behind. Furthermore, IT-linked student challenges, as previously discussed, were related to network coverage, computer literacy and orientation to the e-learning platform. Student and faculty evaluation showed that faculty needed continuous support to cope with the pandemic’s change and impact (Songca 2020c). Support was provided through weekly meetings (discipline and committees) and regular feedback on institutional updates (Songca 2020c). Faculties also needed capacitation in their online assessments, resulting in further university-driven courses for resource strengthening.

In order to bridge the identified challenges and ensure equal opportunities to access learning, each faculty was expected to develop educational resources and catch-up plans for each student who might have been left behind. The university assigned a week to execute the catch-up plans before the semester re-commenced. The *institution offered waivers* to avoid penalising students for what was outside their control but a consequence of the pandemic. This involved lifting duly performance requirements and modifying traditional exams while not compromising the regulatory body’s requirements or standards. Faculties were expected to be flexible and understanding in their approach to students who might have difficulty focusing and experienced challenges beyond their control. For example, students with assessment marks less than 50% or who wanted to improve pass marks received remediation and an opportunity to submit new or revised assignments. At the discretion of the faculty, submission dates were extended, and online learning materials were provided to support the completion of assessments (CHS 2020)

In recognition of the adopted logo of ‘Every Student Matters’, the university recognised the changing academic needs of the students and the *influence of the pandemic on their bio-psycho-social needs* and put countermeasures in place. The campus clinics assumed an active role in their readiness to deal with COVID-19, safeguarding the students’ physical well-being by coordinating COVID-19 tests, liaising with and reporting to the Department of Health, 24/7 availability, which was supportive to the students and faculty alike, administering flu vaccines to all and managing health check-ups (purpose built, daily COVID-19 screening app). In situations where the facilities could not provide PPE, the university provided for the students’ protection. A 4-week psychological first-aid package developed and delivered by faculty addressed the psychological needs of the students.

The time in the clinical skills laboratory was increased to serve more than one goal. The clinical facilities needed to observe COVID-19 regulations and decrease the numbers of students, hence the increased utilisation of the clinical skills laboratory which was permitted by the SANC allowing for additional simulated learning hours (SANC 2020b). Furthermore, the extended time in the clinical skills laboratory allowed for less exposure to the risk of COVID-19 infection, as did the move to online clinical supervision, where students uploaded audio and visual recordings.

### Application of the outcome component of Donabedian’s model

In applying Donabedian’s quality of care model to nursing education in an HEI, during the pandemic, quality nursing education is defined through the triad of Structure, Process and Outcome (Figure 1). The desired outcome of the DHET intervention plans was to save and complete the 2020 academic year (PMG 2020). In line with the HEI’s duty and obligation to provide full support to students, the structure and processes described above ensured the *intended outcome*, and the academic year was salvaged, despite the 3-months’ extension of the academic year. Emergency plans averted the threat to the production of the nursing and midwifery workforce in line with the country’s projected workforce production (ICN 2021).

In addition, the students met the clinical requirements of the regulatory body (SANC) (Baloyi & Mtshali 2018). This is attributed to three factors: firstly, the economical use of time that included moving the academic content to virtual platforms, use of a block system for the theory during the height of the pandemic, as well as the use of simulation in the clinical skills laboratories; secondly, attending to the clinical learning needs of the students through online supervision and catch-up plans, and thirdly, the flexibility in the clinical placement of students, including night duty.

Embracing innovations and educational technologies was an *unintended outcome*. Although innovations and technologies were part of the emergency response tools, they served to fast-track their strategies for online learning. The interruptions to teaching and learning brought by the pandemic provided an opportunity to upskill faculty in Information Communication Technology (ICT) and remote online teaching, virtual clinical support and continuous assessments. In addition, the HEI had an opportunity to divert resources to prioritise establishing or strengthening remote online learning resources. The change led to the provision of other didactic resources, such as simulation and online learning clinical resources, and the development of additional materials such as videos using simulated patients and virtual ward rounds. A further unintended outcome was strengthening partnerships with service and other institutions. In addition, through the preparation of the students with the necessary knowledge, skills, PPE and the provision of support, they were able to adapt and cope in the presence of uncertainty and stress (Jarvis et al. 2021; Mtshali & Jarvis 2021).

## Discussion

The COVID-19 pandemic was unexpected and globally highlighted the unpreparedness of HEIs for the ensuing disaster (Lira et al. 2020), the disruptions and the need to re-vision existing structures, in the process of transitioning to fully online teaching and learning while maintaining professionalism during online engagements (Rabe et al. 2020; UNESCO 2020). Overnight, students and faculty needed to move from traditional face-to-face interactions and rapidly adopt a new decorum (Rabe et al. 2020) while striving for a common outcome.

Donabedian’s model highlights the structural component of the transition to online teaching and learning, and through the process component, it stresses additional challenges experienced by some HEIs. Within South Africa and globally (Agu et al. 2020; Lira et al. 2020), the abrupt transition exposed inequalities among students, with those from rural areas, further challenged in the HEI under discussion by the underpinning principle of ‘leaving no student behind’ (UNESCO 2020). Globally, there were variations in final-year nursing students’ completion times, with 57% of the countries reporting delays in student graduations and 7% of the countries reporting delays of 12 months or more (ICN 2021). This global phenomenon heralds the success of the study setting through its structures and processes in saving the academic year. In further recognition of this outcome, inequalities were exposed of students’ abilities to access education (Agu et al. 2020; Czerniewicz et al. 2020). Against the backdrop of gaps in equality lie reports that students from well-resourced institutions managed to complete their academic programmes within the stipulated time, in contrast to those from resource-constrained institutions who had to extend the length of their programmes to meet all the regulatory requirements (Agu et al. 2020; Czerniewicz et al. 2020). Regulatory bodies adopted different approaches in authorising completion, ranging from allowing an excess of 50% of simulated learning of clinical skills (Mtshali 2021; TBON 2020), higher than the 20% for the HEI under discussion (Baloyi & Mtshali 2018). Like the study setting, the Nursing Board of Iowa (TBON 2020) emphasised the need for catch-up plans.

Fast track was one of the positive unintended outcomes reported by 57% of the National Nurses Associations, which the ICN refers to as one of the biggest gains in the education sector (ICN 2021). Like the HEI under discussion, the COVID-19 pandemic accelerated the adoption of technologies, which required HEIs to develop alternate teaching and learning spaces within a short span of time, adopting blended learning designs or hybrid teaching methods (Lira et al. 2020).

Nursing faculties were not only saddled with transitioning to online teaching and learning but also faced with meeting the requirements of the clinical component of their programmes, challenged by suspension or restrictions in students’ placements in the clinical areas to protect against the risks associated with exposure to COVID-19 (Agu et al. 2021). In some settings, such as Croatia, the focus turned to the theoretical content (Jandric et al. 2020). The HEI under discussion made clinical adjustments and prepared its students with the necessary knowledge, skills and PPE to nurse in a pandemic, demonstrating their ability to adapt (Baloyi & Mtshali 2018) and contributing towards the desired outcome of saving the 2020 academic year, leaving no student behind (PMG 2020).

## Recommendations

Investigate the possibility of HEI continuing with online teaching and learning, reverting to face-to-face teaching and learning strategies or adopting a blended model. Should a blended model be adopted, identify which changed structures and processes from the emergency transition will be retained, and identify the influence of teaching technologies on the teaching and learning process.

## Limitations

The transition of one of the HEIs is examined and discussed, while a country-wide representation offers greater information to global initiatives.

Obtaining data from a single source, of documents only, limited the extraction of data from the academics and students to capture their experiences.

## Conclusion

This report highlighted how the seemingly impossible was made possible in a HEI’s transition to online learning as COVID-19-related interruptions were managed. Before the COVID-19 pandemic, educationists might have argued the impossibility of transitioning, a four-year undergraduate nursing programme in three months, from a traditional approach to fully virtual remote teaching and learning. Critiques might have highlighted the added challenges of transitioning to online teaching and learning, with students predominantly from lower resourced settings and digital migrant faculty. However, despite the critique against the linear nature of Avedis Donabedian’s tripartite model (Ameh et al. 2017), its application has highlighted the influence of the structures and processes on both the intended and unintended outcomes as the 2020 academic year was saved, offering insights for the establishment of guidelines.
